# Supplementary material for: Advance care planning in patients with advanced cancer: A 6-country, cluster-randomised clinical trial
Source: PLoS Med. 2020 Nov 13;17(11):e1003422. doi: 10.1371/journal.pmed.1003422 (PMC7665676; doi:10.1371/journal.pmed.1003422)
Supplement: S1 Text — (DOCX) [file pmed.1003422.s002.docx]

**S1 Text** Inclusion and exclusion criteria ACTION study

| **Inclusion Criteria** | **Exclusion Criteria** |
| --- | --- |
| Histologically confirmed diagnosis of:   - Lung cancer: - small cell - extensive disease/ Stage III or IV* - non-small cell - stage III or IV* - Colorectal cancer: Stage IV or metachronous metastases*   *according to 7th edition of TNM classification and staging system  Written informed consent to participate  WHO performance status of 0-3. | - Age < 18 years - Unable to provide informed consent - Unable to complete questionnaire in country’s language - Less than 3 months anticipated life expectancy - Taking part in a research study that is evaluating palliative care services or communication strategies. |
