# Supplementary material for: Advance care planning in patients with advanced cancer: A 6-country, cluster-randomised clinical trial
Source: PLoS Med. 2020 Nov 13;17(11):e1003422. doi: 10.1371/journal.pmed.1003422 (PMC7665676; doi:10.1371/journal.pmed.1003422)
Supplement: S4 Text — Box: Handling of missing data. Table A: Number and proportion of missing values for sociodemographic and clinical variables, and for the questionnaire items used to build the scores. Table B: Distribution of loss to follow-up at follow-up assessments 1 and 2 by sociodemographic and clinical variables among surviving patients, with p-value from chi-squared test. Table C: Treatment effect on emotional functioning. (DOCX) [file pmed.1003422.s005.docx]

**S4 Text. Supportive information regarding statistical analyses**

**S4 – Box. Handling of missing data**

The data showed a not negligible amount of missing data (Table S1). Data can be assumed to be missing at random (MAR), i.e. the probability of an observation being missing depends only on the observed. In fact, in accord with the Little’s missing completely at random (MCAR) test, data were not MCAR, i.e. the probability of an observation being missing does not depend on both observed and unobserved data (p < 0.001). Moreover, the distribution of lost to follow-up both at follow-up 1 and 2 by socio-demographic and clinical variables among survivals showed significant relationship between the missingness of observations and some variables, e.g. marital status, years of education (Table S2).

Complete-case analysis, that consists in performing the analysis on the subset of subjects with complete information, may lead to biased results unless the missingness mechanism is MCAR or the amount of missing data is negligible.

Multiple imputation, which is based on the MAR assumption, is widely recognised as the most appropriate one in many fields [1-2], and it consists in creating several complete versions of the data by replacing each missing value with more than one plausible value. Each of the resulting complete datasets is then analyzed with standard statistical methods and the results pooled for final inference using the Rubin’s combination rule, to obtain a point estimate and a measure of precision which accounts for uncertainty due to missing information [1]. We created several complete versions of the data by sampling the last *M* iterations after algorithm convergence, choosing *M* according to the rule of thumb based on the average percentage rate of missingness [3].

There are several ways to implement multiple imputation. In this analysis we run a Multivariate Imputation by Chained Equations (MICE) [4-5] with a preliminary step consisting in the selection of a matrix of predictors for each variable with missing values. In particular, we regressed each variable with missing entries on each of the other variables, completed by a preliminary imputation which draw from their empirical marginal distribution, and then selected as predictors the 15 variables which lead to the smallest Akaike’s information criterion values [6]. The marginal regressions used in this preliminary procedure and in the following imputation algorithms varied according to the nature of the outcome variable (linear regression for continuous variables, logistic regression for factor variables with 2 levels, multinomial logistic regression for factor variables with more than 2 levels, and proportional odds models for ordered variables).

The MICE procedure was implemented by using the R package mice [7].

**References**

1. Rubin DB. Multiple Imputation for Nonresponse in Surveys. New York: John Wiley & Sons; 1987.
2. Sterne JA, White IR, Carlin JB, Spratt M, Royston P, Kenward MG, Wood AM, Carpenter JR. Multiple imputation for missing data in epidemiological and clinical research: potential and pitfalls. BMJ. 2009;338:b2393. doi: 10.1136/bmj.b2393.
3. White IR, Royston P, Wood AM. Multiple imputation using chained equations: Issues and guidance for practice. Stat Med. 2011;30:377-99.
4. Raghunathan TE, Lepkowski JM, van Hoewyk J, Solenberger P. A multivariate technique for multiply imputing missing values using a sequence of regression models. Survey Methodology. 2001; 27:85–95.
5. Van Buuren S. Flexible Imputation of Missing Data. Boca Raton, FL: Chapman & Hall; 2012.
6. Li F, Baccini M, Mealli F, Zell ER, Frangakis CE, Rubin DB. Multiple Imputation by Ordered Monotone Blocks with Application to the Anthrax Vaccine Research Program. Journal of Computational and Graphical Statistics. 2014;23;877-892.
7. Van Buuren S, Groothuis-Oudshoorn K. MICE: Multivariate imputation by chained equations. R. Journal of Statistical Software 2011; 45:1-67.

**S4 –Table A. Number and proportion of missing values for sociodemographic and clinical variables, and for the questionnaire items used to build the scores.**

| Sociodemographic variables | | | | Clinical variables | |
| --- | --- | --- | --- | --- | --- |
| variable | N(%) | variable | N(%) | variable | N(%) |
| treat | 0 (0) | children | 15 (1.5) | PB_CancerType | 0 (0) |
| Country | 0 (0) | education | 137 (13.3) | PB_CurrentStage | 3 (0.3) |
| hospital | 0 (0) | sex | 0 (0) | PB_WHOst | 14 (1.4) |
| age | 2 (0.2) | marital | 16 (1.6) | PB_TreatChemo | 4 (0.4) |
| living_with | 26 (2.5) | religious | 20 (1.9) | PB_TreatRadio | 4 (0.4) |
| living_where | 20 (1.9) | minority | 42 (4.1) | PB_TreatImmun | 4 (0.4) |
|  |  |  |  | PB_TreatTarget | 4 (0.4) |
|  |  |  |  | PB_TreatOther | 4 (0.4) |
| Questionnaire items | | | | | |
| baseline | | follow-up 1 | | follow-up 2 | |
| variable | N(%) | variable | N(%) | variable | N(%) |
| PB_PAL01 | 8 (0.8) | PFU1_PAL01 | 228 (22.1) | PFU2_PAL01 | 339 (32.8) |
| PB_PAL02 | 12 (1.2) | PFU1_PAL02 | 237 (23) | PFU2_PAL02 | 338 (32.8) |
| PB_PAL03 | 8 (0.8) | PFU1_PAL03 | 231 (22.4) | PFU2_PAL03 | 339 (32.8) |
| PB_PAL04 | 11 (1.1) | PFU1_PAL04 | 228 (22.1) | PFU2_PAL04 | 338 (32.8) |
| PB_PAL05 | 12 (1.2) | PFU1_PAL05 | 227 (22) | PFU2_PAL05 | 340 (32.9) |
| PB_PAL06 | 10 (1) | PFU1_PAL06 | 226 (21.9) | PFU2_PAL06 | 336 (32.6) |
| PB_PAL07 | 13 (1.3) | PFU1_PAL07 | 226 (21.9) | PFU2_PAL07 | 337 (32.7) |
| PB_PAL08 | 10 (1) | PFU1_PAL08 | 226 (21.9) | PFU2_PAL08 | 336 (32.6) |
| PB_PAL09 | 10 (1) | PFU1_PAL09 | 229 (22.2) | PFU2_PAL09 | 335 (32.5) |
| PB_PAL10 | 12 (1.2) | PFU1_PAL10 | 232 (22.5) | PFU2_PAL10 | 340 (32.9) |
| PB_PAL11 | 11 (1.1) | PFU1_PAL11 | 231 (22.4) | PFU2_PAL11 | 337 (32.7) |
| PB_PAL12 | 10 (1) | PFU1_PAL12 | 232 (22.5) | PFU2_PAL12 | 337 (32.7) |
| PB_PAL13 | 11 (1.1) | PFU1_PAL13 | 229 (22.2) | PFU2_PAL13 | 337 (32.7) |
| PB_PAL14 | 11 (1.1) | PFU1_PAL14 | 234 (22.7) | PFU2_PAL14 | 339 (32.8) |
| PB_EF02 | 11 (1.1) | PFU1_EF02 | 231 (22.4) | PFU2_EF02 | 337 (32.7) |
| PB_EF03 | 12 (1.2) | PFU1_EF03 | 229 (22.2) | PFU2_EF03 | 337 (32.7) |
| PB_EFCAT01 | 10 (1) | PFU1_EFCAT01 | 233 (22.6) | PFU2_EFCAT01 | 337 (32.7) |
| PB_EFCAT02 | 14 (1.4) | PFU1_EFCAT02 | 235 (22.8) | PFU2_EFCAT02 | 339 (32.8) |
| PB_EFCAT03 | 15 (1.5) | PFU1_EFCAT03 | 233 (22.6) | PFU2_EFCAT03 | 336 (32.6) |
| PB_EFCAT04 | 13 (1.3) | PFU1_EFCAT04 | 231 (22.4) | PFU2_EFCAT04 | 339 (32.8) |
| PB_EFCAT05 | 12 (1.2) | PFU1_EFCAT05 | 235 (22.8) | PFU2_EFCAT05 | 337 (32.7) |
| PB_EFCAT06 | 11 (1.1) | PFU1_EFCAT06 | 233 (22.6) | PFU2_EFCAT06 | 338 (32.8) |
| PB_PAL15 | 18 (1.7) | PFU1_PAL15 | 229 (22.2) | PFU2_PAL15 | 337 (32.7) |
| PB_APECC01 | 15 (1.5) | PFU1_APECC01 | 234 (22.7) | PFU2_APECC01 | 339 (32.8) |
| PB_APECC02 | 15 (1.5) | PFU1_APECC02 | 235 (22.8) | PFU2_APECC02 | 337 (32.7) |
| PB_APECC03 | 16 (1.6) | PFU1_APECC03 | 233 (22.6) | PFU2_APECC03 | 339 (32.8) |
| PB_APECC04 | 15 (1.5) | PFU1_APECC04 | 236 (22.9) | PFU2_APECC04 | 338 (32.8) |
| PB_APECC05 | 15 (1.5) | PFU1_APECC05 | 234 (22.7) | PFU2_APECC05 | 339 (32.8) |
| PB_BLFTC01 | 19 (1.8) | PFU1_BLFTC01 | 242 (23.4) | PFU2_BLFTC01 | 344 (33.3) |
| PB_BLFTC02 | 24 (2.3) | PFU1_BLFTC02 | 240 (23.3) | PFU2_BLFTC02 | 344 (33.3) |
| PB_BLFTC03 | 18 (1.7) | PFU1_BLFTC03 | 242 (23.4) | PFU2_BLFTC03 | 341 (33) |
| PB_BLFTC04 | 20 (1.9) | PFU1_BLFTC04 | 239 (23.2) | PFU2_BLFTC04 | 343 (33.2) |
| PB_PATSAT01 | 14 (1.4) | PFU1_PATSAT01 | 240 (23.3) | PFU2_PATSAT01 | 355 (34.4) |
| PB_PATSAT02 | 12 (1.2) | PFU1_PATSAT02 | 242 (23.4) | PFU2_PATSAT02 | 358 (34.7) |
| PB_PATSAT03 | 10 (1) | PFU1_PATSAT03 | 242 (23.4) | PFU2_PATSAT03 | 361 (35) |
| PB_PATSAT04 | 38 (3.7) | PFU1_PATSAT04 | 268 (26) | PFU2_PATSAT04 | 382 (37) |
| PB_PATSAT05 | 46 (4.5) | PFU1_PATSAT05 | 272 (26.4) | PFU2_PATSAT05 | 382 (37) |
| PB_PATSAT06 | 41 (4) | PFU1_PATSAT06 | 266 (25.8) | PFU2_PATSAT06 | 383 (37.1) |
| PB_PATSAT07 | 25 (2.4) | PFU1_PATSAT07 | 260 (25.2) | PFU2_PATSAT07 | 378 (36.6) |
| PB_COPE01 | 25 (2.4) | PFU1_COPE01 | 253 (24.5) | PFU2_COPE01 | 355 (34.4) |
| PB_COPE02 | 17 (1.6) | PFU1_COPE02 | 239 (23.2) | PFU2_COPE02 | 346 (33.5) |
| PB_COPE03 | 21 (2) | PFU1_COPE03 | 245 (23.7) | PFU2_COPE03 | 349 (33.8) |
| PB_COPE04 | 23 (2.2) | PFU1_COPE04 | 247 (23.9) | PFU2_COPE04 | 351 (34) |
| PB_COPE05 | 22 (2.1) | PFU1_COPE05 | 247 (23.9) | PFU2_COPE05 | 349 (33.8) |
| PB_COPE06 | 25 (2.4) | PFU1_COPE06 | 249 (24.1) | PFU2_COPE06 | 349 (33.8) |
| PB_COPE07 | 21 (2) | PFU1_COPE07 | 249 (24.1) | PFU2_COPE07 | 347 (33.6) |
| PB_COPE08 | 13 (1.3) | PFU1_COPE08 | 244 (23.6) | PFU2_COPE08 | 349 (33.8) |
| PB_COPE09 | 21 (2) | PFU1_COPE09 | 250 (24.2) | PFU2_COPE09 | 354 (34.3) |
| PB_COPE10 | 19 (1.8) | PFU1_COPE10 | 247 (23.9) | PFU2_COPE10 | 351 (34) |
| PB_COPE11 | 17 (1.6) | PFU1_COPE11 | 254 (24.6) | PFU2_COPE11 | 352 (34.1) |
| PB_COPE12 | 22 (2.1) | PFU1_COPE12 | 250 (24.2) | PFU2_COPE12 | 350 (33.9) |
| CAT_BL | 6 (0.6) | CAT_FU1 | 226 (21.9) | CAT_FU2 | 333 (32.3) |

**S4 – Table B. Distribution of lost to follow-up 1 and 2 by socio-demographic and clinical variables among survivals with p-value from chi-2 test.**

|  | follow-up 1 | | | follow-up 2 | | |
| --- | --- | --- | --- | --- | --- | --- |
|  | observed, N(%) | missing, N(%) | p | observed, N(%) | missing, N(%) | p |
| Gender |  |  |  |  |  |  |
| males | 126 (56.6) | 498 (61.6) |  | 154 (61.85) | 421 (59.97) |  |
| fenales | 97 (43.5) | 311 (38.44) | 0.172 | 95 (38.15) | 281 (40.03) | 0.603 |
| Marital status |  |  |  |  |  |  |
| married | 139 (62.3) | 571 (50.6) |  | 157 (63.05) | 497 (70.8) |  |
| not marreid/widower/divorced | 78 (35) | 228 (28.2) | 0.033 | 87 (34.94) | 195 (27.78) | 0.075 |
| Country |  |  |  |  |  |  |
| BE | 54 (24.22) | 126 (15.57) |  | 62 (24.9) | 104 (14.81) |  |
| SI | 15 (6.73) | 70 (8.65) |  | 13 (5.22) | 57 (8.12) |  |
| IT | 64 (28.7) | 98 (12.11) |  | 58 (23.29) | 96 (13.68) |  |
| UK | 47 (21.08) | 189 (23.36) |  | 62 (24.9) | 152 (21.65) |  |
| NL | 28 (12.56) | 210 (25.96) |  | 30 (12.05) | 193 (27.49) |  |
| DK | 15 (6.73) | 116 (14.34) | <0.001 | 24 (9.64) | 100 (14.25) | <0.001 |
| Cancer type |  |  |  |  |  |  |
| Small cell - extensive disease lung cancer | 28 (12.56) | 75 (9.27) |  | 34 (13.65) | 58 (8.26) |  |
| Non-small cell lung cancer | 103 (46.19) | 345 (42.65) |  | 121 (48.59) | 288 (41.03) |  |
| Colon cancer | 69 (30.94) | 285 (35.23) |  | 68 (27.31) | 266 (37.89) |  |
| Rectal cancer | 23 (10.31) | 104 (12.86) | 0.236 | 26 (10.44) | 90 (12.82) | 0.002 |
| Stage of cancer |  |  |  |  |  |  |
| Stage III lung cancer | 28 (12.56) | 99 (12.24) |  | 37 (14.86) | 76 (10.83) |  |
| Satge IV lung cancer | 106 (47.53) | 321 (39.68) |  | 120 (48.19) | 271 (38.6) |  |
| Stage IV colorectal cancer | 65 (29.15) | 243 (30.04) |  | 64 (25.7) | 225 (32.05) |  |
| Metachronous metast colrectal cancer | 24 (10.76) | 143 (17.68) | 0.07 | 27 (10.84) | 128 (18.23) | 0.004 |
| WHO performance status |  |  |  |  |  |  |
| <=2 | 186 (83.4) | 713 (88.1) |  | 206 (82.73) | 631 (89.89) |  |
| >2 | 35 (15.7) | 84 (10.4) | 0.075 | 41 (16.47) | 60 (8.55) | 0.002 |
| Living with |  |  |  |  |  |  |
| spouse/partner | 150 (67.26) | 589 (72.81) |  | 169 (67.87) | 509 (72.51) |  |
| alone | 68 (30.49) | 199 (24.6) | 0.203 | 73 (29.32) | 176 (25.07) | 0.381 |
| Living where |  |  |  |  |  |  |
| private household | 203 (91.03) | 755 (93.33) |  | 226 (90.76) | 658 (93.73) |  |
| institution | 2 (0.9) | 4 (0.49) |  | 3 (1.2) | 3 (0.43) |  |
| other | 14 (6.28) | 34 (4.2) | 0.525 | 14 (5.62) | 27 (3.85) | 0.33 |
| Children |  |  |  |  |  |  |
| yes | 191 (85.7) | 695 (85.9) |  | 218 (87.55) | 597 (85.04) |  |
| no | 29 (13) | 102 (12.7) | 0.977 | 28 (11.24) | 93 (13.25) | 0.603 |
| Religious |  |  |  |  |  |  |
| yes | 125 (56.05) | 377 (46.6) |  | 132 (53.01) | 328 (46.72) |  |
| no | 57 (25.56) | 324 (40.05) |  | 72 (28.92) | 283 (40.31) |  |
| prefer not specify | 34 (15.25) | 95 (11.74) | 0.001 | 39 (15.66) | 77 (10.97) | 0.01 |
| Minority |  |  |  |  |  |  |
| yes | 1 (0.45) | 7 (0.87) |  | 2 (0.8) | 6 (0.85) |  |
| no | 212 (95.07) | 770 (95.18) | 0.774 | 238 (95.58) | 666 (94.87) | 0.9 |
| Age* | 66.6 (0.65) | 66.1 (0.34) | 0.4275 | 66.6 (0.65) | 65.9 (0.37) | 0.3547 |
| Years of education* | 12.1 (0.39) | 13.3 (0.17) | 0.0029 | 12.43 (0.35) | 13.38 (0.18) | 0.0116 |

* p-value from t-test.

**S4 – Table C. Treatment effect on emotional functioning.**

Results from linear multilevel model with a causal intercept and with hospital as random level adjusting for baseline level of the score, for Country, years of education, WHO performance status, and having children. The analyses are performed on imputed data (M=37) on survivals to follow-up 1 (N=1032, 85 deaths before follow-up1) for analyses on follow-up1 and on survivals to follow-up 2 (N=951, 166 deaths before follow-up2) for analyses on follow-up2.

|  | **Follow-up 1**  N=1032 | | **Follow-up 2**  N=951 | |
| --- | --- | --- | --- | --- |
| **Variable** | **Coefficient** | **p-value** | **Coefficient** | **p-value** |
| Treatment (not treated ref.) |  |  |  |  |
| treated | -0.024 | 0.666 | -0.076 | 0.414 |
| Baseline value of emotional functioning | 0.608 | <0.001 | 0.613 | <0.001 |
| Years of education | 0.014 | 0.023 | 0.001 | 0.873 |
| Country (Belgium ref.) |  |  |  |  |
| Slovenia | 0.115 | 0.337 | 0.024 | 0.905 |
| Italy | 0.044 | 0.647 | -0.002 | 0.990 |
| United Kingdom | 0.165 | 0.053 | -0.009 | 0.961 |
| Netherlands | 0.257 | 0.002 | 0.007 | 0.964 |
| Denmark | 0.279 | 0.004 | -0.032 | 0.865 |
| WHO performance status (0 –ref.) |  |  |  |  |
| 1 | -0.133 | 0.020 | -0.094 | 0.275 |
| 2 | -0.295 | 0.002 | -0.244 | 0.123 |
| 3 | -0.887 | <0.001 | -1.162 | 0.022 |
| Having children (no children ref.) |  |  |  |  |
|  | 0.103 | 0.191 | 0.141 | 0.235 |
