# Supplementary material for: Advance care planning in patients with advanced cancer: A 6-country, cluster-randomised clinical trial
Source: PLoS Med. 2020 Nov 13;17(11):e1003422. doi: 10.1371/journal.pmed.1003422 (PMC7665676; doi:10.1371/journal.pmed.1003422)
Supplement: Attachment — Submitted filename: REVIEW PMEDICINE ACTION Trial REVISION.pdf [file pmed.1003422.s007.pdf]

REVIEW:

Advance care planning in patients with advanced cancer; a six country, clusterrandomized clinical trial

PMEDICINE-D-20-00632_R2

(Please note: there was a R1- and a R2-version of the revision, and only the latter contained the reply to the reviewers; I did not understand why this first revision is numbered R2).

Thanks to the authors for considering many of my and the other reviewers‘ suggestions. In particular, I am very happy that the authors have changed „alternative“ to „additional“ in their last sentence.

In my eyes, however, some of the critical items have not been appreciated sufficiently:

R1.1 I wrote in my review:

A. **Reasons related to the intervention**

…

2. There is another important influential factor that warrants discussion. Implementing an ACP program such as Respecting Choices should not be limited to establishing a conversation process between facilitator, patient, and significant others; besides this personal level, there is also an institutional and regional level that logically must be considered if the ultimate goal of ACP that patients‘ preferences are known and honored is to be achieved.
In particular, Respecting Choices is known to have always pushed both levels, the „systems change“ level (institutional / organisational and regional development), and the personal conversation level. The ACTION trial has limited the intervention on the mere personal level. This needs to be discussed here: Why was the decision made? What were the personal, institutional and regional barriers observed in the respective countries? Are there any further indications that a „systems change“ would have been necessary to let the intervention develop a positive impact on the clinical encounters?

The authors reply,

Implementation at institutional and regional level as well as at the patient level is typical for the Respecting Choices approach. We acknowledged from the start of the ACTION project that, given the context of a clinical trial, we were not able to implement the intervention at the institutional and regional level: ACP conversations were offered only to trial participants. We could therefore not e.g. advertise in e.g. elevators or waiting rooms. This decision was thus based on methodological barriers. We indicated in the discussion that, as a result, the programme might not have been sufficiently adapted to local circumstances and needs. We now added that this was especially true for countries not familiar with ACP, such as Denmark, Slovenia and Ital,y and that the programme was therefore probably notfullyv integrated with routine services which may have reduced its effect

and add to their discussion (p17) the sentence:

…, especially in countries not familiar with ACP, such as Denmark, Slovenia and Italy. Therefore the programme was probably not integrated with routine services which may have reduced its effect.

However, this amendment misses the decisive point. The authors concede very clearly and correctly, that „implementation at institutional and regional level … is typical for the Respecting Choices approach." The reason why RC has chosen to push institutional and regional implementation of ACP with the same determination as the ACP conversation itself is, however, that **ACP is not likely to be effective** without what RC calls a systems change at institutional and regional levels. Thus, it is not only „countries not familiar with ACP" that require regional and institutional implementation of ACP in order for it to become effective; it is every group that undertakes to implement ACP at all, including RC itself in the La Crosse region. At a national ACP conference in La Crosse in autumn 2016, RC staff reported that for various reasons the La Crosse coordinator position had been vacant for more than a year („ACP coordinator being the role that ensures institutional and regional coordination). As a consequence, even a program as well-established as RC in La Crosse itself for more than 20 years immediately and notably lost effect, according to the report.

What I am saying is: From the perspective that ACP requires institutional and regional implementation, the ACTION trial's intervention was never likely to become effective because it lacked two out of three essential components. I understand that for the authors this is a difficult position to report, and the authors do not have to share this view, but since they cooperated with Respecting Choices, and report a negative result, in my eyes it is inevitable to make this possible severe objection clear and transparent in their discussion. Limiting the objection to „countries not familiar with ACP", in contrast, not only misses but obscures the point.

R1.2 I wrote in my review:

B. Reasons related to the Study Design

…

1. …
2. But also with regard to clinical outcomes like hospitalisation: Is ACP likely to change the course of patients with advanced, non-curable cancer? Advance Care Planning, as the authors rightly quote from the EAPC White Paper, allows individuals to reflect, to plan and to document their preferences for future critical treatment decisions that may need to be taken when the now planning individual has become incapable of decision making. Any clinical effect of ACP, then, is to be expected (only) if patients are rendered incompetent at some time in the course of their illness AND if at that time medical decisions have to be taken for which the preferences planned and documented in advance can be regarded relevant. In advanced cancer, however, it would not be unexpected that most treatment decisions are made with the competent patient, while these patients become incompetent typically (i.e., with few exceptions) in the course of their dying process when any further attempts to sustain life would be futile and therefore medically not indicated (justifiable). Therefore, it would be interesting to learn whether the authors have collected any quantitative or qualitative impressions on whether medical treatment decisions had to be taken at all with study patients incapable of decision making so that an advance care plan, if present, could have become relevant. In any case, it ought to be discussed whether ACP is likely to make an effect in this patient group – unless there is an assumption that ACP indirectly also transforms care planning, i.e. the medical decision making between oncologists and competent, actively

c. participating patients. Such an assumption, however, should be made transparent in the discussion.

The authors reply,

Indeed, ACP is considered to enable individuals to reflect, discuss, and document preferences for future treatment and care. For a number of reasons we think this process could affect the decision-making process of patients with advanced cancer. 1. ACP is conceptualised as to be especially useful for situations in which the individual has become incapable of decision making. This can also include decision-making while being in pain or in distress, as is known to be common in patients with advanced cancer. 2. The process of reflecting and discussing preferences for care can also prepare and empower patients for decisions they make themselves, while they are competent. We do not have data about whether medical treatment decisions had to be taken with participants incapable of decision-making. We now added an assumption about the potential of ACP interventions to transform care planning.

and add in their introduction:

ACP interventions have the potential to prepare patients for decision-making when they are unable to make their own decisions; moreover they have the potential to empower patients to engage in in-the-moment decisionmaking (2).

Again, I have to say that I am not satisfied with this reply. My point was that ACP because of its very nature and purpose is not likely to be effective in advanced cancer. When patients with advanced cancer deteriorate, there is usually ample time and opportunity to discuss the remaining options for live-sustaining treatment with them – as long as there are any. I am surprised to read that instances where ACP should become effective may also be when patients with advanced cancer are „in pain or in distress" – this statement is not based on evidence, and it is not at all plausible. When a patient with advanced cancer is in agony, then this is certainly not the time to chose between life-sustaining versus palliative treatment options. Oncologic treatment options such as operation, chemotherapy or radiation almost always presuppose consent of a competent individual, and they will always receive what is regarded necessary for an informed consent. As a consequence, any prior ACP, while it may or may not have some preparatory effect (second argument of the authors), will usually not (with rare exceptions) guide medical decision making on whether or not to use life-sustaining measures when patients with advance cancer deteriorate. It is possible, and even likely, that whether patients with advanced cancer are comfortable and enjoy best possible quality of life strongly relates to how oncological treatment options are offered to them in the sense of an optimal process of shared decision making, and also whether they are offered optimal palliative care from early on, but not so much ACP. That ACP may prepare for discussions of oncologic treatment is an interesting hypothesis that has no evidence base for this special patient population, certainly not in the paper of Rebecca Sudore et al. quoted by the authors (reference #10).

In my eyes it should be discussed in the limitation section that the ACTION trial may have ended with a negative result because ACP may not be effective in changing quality of life and treatment decisions in the population of patients with advanced cancer since these rarely require treatment decisions to be based on advance care planning.

R1.3 I wrote in my review:

C. „The European context"

> … Given the alternative good possible explanations reflected above for the negative results found in this study, and the strong body of evidence for overdiagnosis and -treatment (and lack of patient-centred care) also in many European countries, this paper does not seem the right place to speculate whether ACP in general may not be a necessary or useful tool for Europe altogether.

The authors now write in their paper:

Fourthly, there might be relatively little to gain from ACP in Europe. Some studies suggest that the US may be more prominent in its use of expensive, resource-intensive services at the end of life, such as ICU care (48). Hence, ACP might be more effective in the US to counteract potentially futile treatment. This hypothesis warrants further study. Moreover, to a certain extent, ACP may have been part of usual care in some of the countries, which may have further limited the effects of the intervention

I remain very unhappy and somewhat upset (confer also comment # 22 of Reviewer # 3) with the now remaining assertion that „ACP (sic!) might be more effective in the US [than in Europe] to counteract potentially futile treatment“. In the light of what can be said about possible shortcomings of the study design (including intervention and target population), this is not a „hypothesis that warrants further study“ but a hypothesis that does not warrant to be made here. Again, we are talking about ACP for patients with advanced cancer, i.e. the very target population of all for whom the lowest effect of ACP is to be expected, for the reasons given above. Besides (and not mentioned in my first review), one of the strongest (and finest) pieces of evidence of ACP was a randomised controlled trial of aged patients in an Australian hospital (Detering et al, BMJ 2010) – with Australia being certainly more anglosaxon rather than US American. My suggestion, therefore, is to dismiss the US-European comparison; if the authors remain not ready to do so, than the least would be to confine their statement to the special patient population they have studied, i.e. with advanced cancer.
