# Supplementary material for: Advance care planning in patients with advanced cancer: A 6-country, cluster-randomised clinical trial
Source: PLoS Med. 2020 Nov 13;17(11):e1003422. doi: 10.1371/journal.pmed.1003422 (PMC7665676; doi:10.1371/journal.pmed.1003422)
Supplement: Attachment — Submitted filename: Reply to the editors&reviewers_ACTION_FINAL.docx [file pmed.1003422.s006.docx]

| REVIEWERS” AND EDITORS” FEED BACK | AUTHORS’ RESPONSE | EDITS TO THE MANUSCRIPT |
| --- | --- | --- |
| **EDITORS’ FEEDBACK** |  |  |
| *Please structure your abstract using the PLOS Medicine headings (Background, Methods and Findings, Conclusions) - "methods and findings" is a single subsection. | We now adapted the abstract as indicated. | The headings ‘Patients and methods’ and ‘Results’ have been replaced by the heading ‘Methods and findings’. |
| *At this stage, we ask that you include a short, non-technical Author Summary of your research to make findings accessible to a wide audience that includes both scientists and non-scientists. The Author Summary should immediately follow the Abstract in your revised manuscript. | We added an Author Summary to our manuscript. | See page 4 for our Author Summary. |
| *In the Abstract, it would be good to include a (short) statement of what the provision was for the Control arm, so the distinction between intervention and control can be understood. | We now indicated more clearly the distinction between intervention and control arms. | *Abstract*:  The ACTION Respecting Choices ACP intervention, as offered to patients in the intervention arm, included scripted ACP conversations between patients, family members and certified facilitators; standardized leaflets and standardized advance directives. Control patients received care as usual. |
| *In the last sentence of the Abstract Methods and Findings section, please include a brief summary of any of the key limitation(s) of the study's methodology. | We included a key limitation of the study in our abstract. | *Abstract*:  A key limitation of the study is that recruitment rates were higher in control than in intervention hospitals. |
| *It would be good to update the intext referencing callout at this stage if you can - please change to sequential numbers in square brackets (eg, [1, 2] etc) rather than superscript. If using referencing software this should be quick and easy. | We now adapted the intext referencing as requested. |  |
| *It would be important to robustly address the concerns raised by reviewer 3, particularly as this reviewer raises a number of points regarding the reliability of the analyses and whether some of the major conclusions drawn in the paper are indeed driven by the data. | We thoroughly addressed the concerns of reviewer 3 as requested. |  |
|  |  |  |
| **Comments from the reviewers** |  |  |
| **Reviewer #1: Alex McConnachie** |  |  |
| The following is a statistical review of Korfage et al's report on ACTION, an international, multicentre, cluster randomised trial of the use of advanced care planning for patients with advanced lung and colorectal cancer. Overall, I found the paper to be interesting, and well written. I do have a few comments, but these are generally quite minor. | We thank the reviewer for these kind words. |  |
|  |  |  |
| The cluster randomised trial design is fully appropriate for this intervention, due to the likelihood of contamination. The problem is that participants appear to have known which arm of the trial they were being recruited into, given the very different consent rates (29 vs. 56%) in the two arms. Potentially, two quite different groups of patients may have been recruited into the trial. According to Table 1, the differences do not seem too extreme, plus the analysis includes plenty of adjustments, but with such different recruitment rates, there will always be the doubt that something could be biasing the overall results.  Had participants been asked for consent, and provided baseline measurements, before being told whether their hospital was in the ACP or control arm, this might have allowed some assessment of this differential engagement with the trial. Ideally, one would ask whether the factors associated with participation are the same in ACP hospitals and control hospitals. Is there any information about those who did not take part that could be used in this way? | Participants knew which arm they were being recruited into. We agree with the reviewer that the differences in consent rates suggest that knowledge about being recruited into the control or the intervention arm may have affected the willingness to participate.  This was addressed under the limitations in the discussion in the original manuscript as follows:  *First, that fewer eligible patients in the intervention group were asked to participate than in the control group (66% versus 84%) suggests some level of gatekeeping (35). In addition, recruitment rates were higher in control than in intervention hospitals. These factors may have resulted in unmeasured baseline differences between the study groups.*  We were not allowed by research ethics committees (RECs) to collect information on reasons of eligible patients who decided not to take part. Hospital staff suggested that the need to have ACP conversations was perceived as burdensome by patients who rejected participation in the intervention arm and ‘only’ having to complete questionnaires was a reason to accept participation in the control arm. |  |
|  |  |  |
| I was able to replicate the sample size calculation from the information provided, but given that the detectable between-group difference was given a half a standard deviation, it is not clear why the required sample size should reduce when it was decided to change to a different primary outcome. | In calculating whether the required sample size could be adapted when changing from the original primary outcome (EF4) to the new one (EF10) we did the following:  We used the method of known-groups validation to evaluate the relative validity (i.e., statistical power to detect group differences) of the EF10 compared with the EF4 (Fayers, Machin, 2016). For each patient characteristic and for each questionnaire item (except for the emotional functioning items), participants were divided into 'known groups'; two groups based on a priori formulated expectations on differences with regard to emotional functioning (EF). For example, we compared the EF scores of patients with lung cancer with the EF scores of patients with colorectal cancer. In case of continuous variables, such as age, the median value was used as a cut-off for the dichotomization. If the median was similar to the highest or the lowest score, and thus no groups could be created based on the median value, we calculated the mean value and used it as the cut-off to create two groups. In total, we formed 53 pairs of known-groups for comparison.  We used the two-sample t-test (assuming equal variances) to assess significant differences between the known-groups for the comparison. The relative validity was only calculated when the t-test for at least one of the measures being compared (EF10 or EF4) showed a significant difference (p<0.05) (Fayers, Machin, 2016). For each comparison the t-statistic was calculated and subsequently squared. Using the squared t-statistic, the ratio (= relative validity) was calculated for each comparison by dividing the squared t-statistic of e.g. the EF10 by the squared t-statistic of EF4. The mean relative validity across all characteristics or variables was calculated with a bootstrap based 95% confidence interval. A mean relative validity above 1 indicates that the EF10 has higher relative validity than the EF 4. With increasing relative validity, one can expect more power gained by using the EF10. Based on the mean relative validity we estimated the potential savings in sample size requirements using the EF10 compared with using EF4.  We hope this clarifies our approach. For more details, we kindly refer to our publication on this approach in Jabbarian et al, 2019, Value in Health. |  |
|  |  |  |
| The analyses are perfectly acceptable. However, in the title of Table 2, the term "causal intercept" was not one I have encountered before (and an internet search did not shed any light). This may need some additional explanation. Another point on reading Table 2, is that it is not obvious whether positive and negative changes for each outcome represent an improvement or a deterioration. Also, though the presentation of the results is generally good, the ICCs for the different outcomes are not reported, as recommended by CONSORT. | To avoid confusion we now removed this term.  We now indicate in Table 2 per outcome whether positive and negative changes indicate improvement or deterioration.  We now also report the ICCs. | See Table 2. |
|  |  |  |
| **Reviewer #2:** |  |  |
| Korfage and colleagues report the results of a large cluster-randomised controlled trial to examine the effect of implementing an ACP program in hospitalised patients with advanced cancer in six European countries. Intervention and control group showed no difference with regard to quality of life, the primary outcome, nor with most of the secondary outcomes including hospitalisation rate. Also in the intervention group, very few (even though more than in the control group) of the patients had an AD in their hospital charts.  Congratulation to the authors for this important paper that to me seems methodologically sound, and well written. I have only few and minor suggestions for the sections background, methods and results (see below). I have, however, a major concern that relates to the interpretation of the „negative" findings in the discussion section: | We thank the reviewer for these positive words and the careful considerations about our study. |  |
|  |  |  |
| The authors write: „The limited effectiveness of the complex ACP intervention evaluated in this study may be explained by 1) characteristics of the intervention, 2) patients' preferences regarding ACP and Ads or 3) the European context."  In fact there are a number of further possible explanations why this study showed no effect, and given the high attention this large and thoroughly done study is likely to attract, and the possible large impact of reporting a negative outcome in this relatively new and highly sensitive field, it is certainly warranted that these explanations are discussed in detail. In particular, a negative finding of a methodologically sound trial examining the effect of an instrument that has so strong merits as ACP may well cause the authors to take a step back and reconsider their assumptions and design. In my eyes, other passages in the manuscript can and should be shortened (for example the first 8 lines of the background) in order to allow for a thorough critical appraisal of the reasons why this study may have shown no effects. Here is my suggestion of the reasons that ought to be considered: | We agree with the reviewer that a critical appraisal of our results is indicated. We have now expanded the set of explanations, see details below, and shortened other texts, for instance the first six lines of the background. |  |
|  |  |  |
| A. Reasons related to the intervention |  |  |
| 1. The authors correctly report that they could not ensure intervention delivery. It would be worthwhile to make this relevant consideration become somewhat more prominent, for example by repeating the corresponding key results.  a. First of all, it seems that conversations as the core of the intervention did take place most of the time (90%), occurred according to protocol (86%), they were extensive in time (93 minutes), contrary to most recommendations, though, they comprised rarely more than one encounter (1.3).  b. However, only 41% of the patients who had ACP conversations proceeded to signing an advance care plan. This is very unusual. Do the authors have any hints from the process evaluation why this was the case? It would be helpful to understand why so many patients did not sign written advance care plans in order to appreciate how much of the intervention was in fact delivered. If it turns out that for practical or cultural reasons, the intervention was insufficiently realised, then this is of central importance for the interpretation of the results and needs to find entry also in the conclusion. | 1. Indeed, intervention delivery is a common concern in trials of communication interventions. We therefore carefully and closely monitored fidelity to the intervention protocol, using a specifically developed, standardized procedure. Fidelity to the intervention appeared to be good.  a. The number of patients with one encounter only was relatively high. This may have affected the impact of the ACP program. We now mention this in the discussion.  b. Only 147 out of 396 patients (37%) who had ACP conversations handed in a signed copy of an advance directive to the facilitator. This means that at least 37% of patients completed an advance directive. This does not necessarily mean that the intervention was not fully implemented, because completing an advance directive and sharing it with the facilitator was optional. | *Discussion, page 16:* Further, the number of ACP conversations was limited to an average of 1.3 per patient. This may have affected the impact of the ACP program. |
|  |  |  |
| 2. There is another important influential factor that warrants discussion. Implementing an ACP program such as Respecting Choices should not be limited to establishing a conversation process between facilitator, patient, and significant others; besides this personal level, there is also an institutional and regional level that logically must be considered if the ultimate goal of ACP that patients' preferences are known and honored is to be achieved.  In particular, Respecting Choices is known to have always pushed both levels, the „systems change" level (institutional / organisational and regional development), and the personal conversation level. The ACTION trial has limited the intervention on the mere personal level. This needs to be discussed here: Why was the decision made? What were the personal, institutional and regional barriers observed in the respective countries? Are there any further indications that a „systems change" would have been necessary to let the intervention develop a positive impact on the clinical encounters? | Implementation at institutional and regional level as well as at the patient level is typical for the Respecting Choices approach. We acknowledged from the start of the ACTION project that, given the context of a clinical trial, we were not able to implement the intervention at the institutional and regional level: ACP conversations were offered only to trial participants. We could therefore not e.g. advertise in e.g. elevators or waiting rooms. This decision was thus based on methodological barriers.  We indicated in the discussion that, as a result, the programme might not have been sufficiently adapted to local circumstances and needs. We now added that this was especially true for countries not familiar with ACP, such as Denmark, Slovenia and Ital,y and that the programme was therefore probably notfullyv integrated with routine services which may have reduced its effect. | *Discussion, page 17:* …, especially in countries not familiar with ACP, such as Denmark, Slovenia and Italy. Therefore the programme was probably not integrated with routine services which may have reduced its effect. |
|  |  |  |
| B. Reasons related to the Study Design |  |  |
| If a study so well done as this one, and testing such an established intervention, does not find the expected group difference, it is warranted to reconsider whether the expectation was legitimate in the first place, for example asking the following questions: |  |  |
| 1. Does improvement of quality of life belong to the goals that ACP is intended to reach? The authors correctly state that the ultimate goal of ACP is that patients' preferences are known and honoured when medical decisions have to be made. While I appreciate that relying on the medical team to know my well reflected treatment preferences for such a case may cause a certain sense of reassurance, at least immediately after the planning, personally I am having a hard time to understand how ACP could possibly exert a long-term effect on quality of life. Also, I am not aware of any strong evidence that this is likely to happen. I suggest, therefore, for the authors to discuss whether - in retrospect - the chosen primary (and related secondary) outcomes could really be expected to be affected by ACP even if optimally delivered and implemented. | Quality of life is a multi-dimensional concept, entailing physical, emotional and social aspects. Our primary outcome is emotional functioning, as assessed with a subscale of the EORTC-QLQ-C30 quality of life scale.  When opting for emotional functioning as the primary outcome measure of ACTION, we reasoned that an increased awareness of patients that their preferences matter, and that sharing these with relatives and healthcare professionals and knowing they would be taken into account would be very reassuring for seriously ill people.  Quality of life has more often been reported as outcome measure in randomized clinical trials on ACP in patients with advanced cancer. As we report in the introduction section, the results of these studies have been conflicting.  In the EAPC white paper on advance care planning, a Delphi panel did not consider quality of life a suitable outcome measure to assess the effects of advance care planning (Rietjens et al, 2017, the Lancet Oncology).  Therefore, in hindsight, we may have opted for a different primary outcome measure. We now added these reflections to the manuscript. | *Discussion, page 17-18:* Secondly, the choice and timing of outcome measurements may have been suboptimal. Our primary outcome measure was quality of life, operationalized by the EORTC emotional functioning items, at 11-12 weeks of follow-up. In 2017, a large international Delphi panel agreed that quality of life is not the most appropriate outcome of ACP (6). Goal concordant care might have been a better primary outcome, but is very difficult to measure because a validated measure to assess goal-concordant care is lacking (1). Effects of ACP could also predominantly occur in the relational domain: patients and relatives may indeed have been supported to discuss and exchange views about values, goals and preferences, but this outcome was not explicitly assessed in our trial. The qualitative data as collected in the ACTION trial may shed some light on these potential effects. Although we checked medical files up until death for some patients, the timing of our outcome measurement may have been suboptimal: effects may occur at a later stage for at least some patients, at a time when their situation deteriorates and actual decisions have to be made. |
|  |  |  |
| 2. But also with regard to clinical outcomes like hospitalisation: Is ACP likely to change the course of patients with advanced, non-curable cancer? Advance Care Planning, as the authors rightly quote from the EAPC White Paper, allows individuals to reflect, to plan and to document their preferences for future critical treatment decisions that may need to be taken when the now planning individual has become incapable of decision making. Any clinical direct effect of ACP, then, is to be expected (only) if patients are rendered incompetent at some time in the course of their illness AND if at that time medical decisions have to be taken for which the preferences planned and documented in advance can be regarded relevant. In advanced cancer, however, it would not be unexpected that most treatment decisions, even when approaching the very end of life, are made with the competent patient, while these patients become incompetent typically (i.e., with few exceptions) only late in the course of their dying process when any further attempts to sustain life would be futile and therefore medically not indicated (justifiable). Therefore, it would be interesting to learn whether the authors have collected any quantitative or qualitative impressions on whether medical treatment decisions had to be taken at all with study patients incapable of decision making so that an advance care plan, if present, could have become relevant. In any case, it ought to be discussed whether ACP is likely to make an effect in this patient group - unless there is an assumption that ACP indirectly also transforms care planning, i.e. the medical decision making between oncologists and competent, actively participating patients. Such an assumption, however, should also be made transparent in the discussion. | Indeed, ACP is considered to enable individuals to reflect, discuss, and document preferences for future treatment and care.  For a number of reasons we think this process could affect the decision-making process of patients with advanced cancer.  1. ACP is conceptualised as to be especially useful for situations in which the individual has become incapable of decision making. This can also include decision-making while being in pain or in distress, as is known to be common in patients with advanced cancer.  2. The process of reflecting and discussing preferences for care can also prepare and empower patients for decisions they make themselves, while they are competent.  We do not have data about whether medical treatment decisions had to be taken with participants incapable of decision-making.  We now added an assumption about the potential of ACP interventions to transform care planning. | *Introduction, page 5:*  ACP interventions have the potential to prepare patients for decision-making when they are unable to make their own decisions; moreover they have the potential to empower patients to engage in in-the-moment decision-making (2). |
|  |  |  |
| C. „The European context" |  |  |
| Given the strong case that the intervention may not have reached the actual decision making process (as indicated by a low rate of written advance care plans, and by the omisson of any institutional and regional implementation efforts in the sense of a probably necessary change management), and / or that the intervention in its nature may not be suitable to change many of the chosen outcomes, or unfold a relevant effect for the chosen patient population even if perfectly delivered and optimally measured, I would advise to reconsider the „European" argument. Here, the authors generalise their findings to ACP in general, suggesting that in Europe (i.e., for all patient populations in all European countries), there may be a lesser need for ACP because of a lesser rate of over-treatment, compared with the U.S. This is a far-reaching interpretation that in my perception is not warranted by the design nor by the results of this study. Given the alternative good possible explanations reflected above for the negative results found in this study, and the strong body of evidence for both overdiagnosis and overtreatment (and lack of patient-centred care) also in many European countries, this paper does not seem the right place to speculate whether ACP in general may not be a necessary or useful tool for European patients altogether. | We agree with the reviewer that we may have been somewhat firm in our reflections on the potentially lesser need of ACP in Europe. We have now adapted this section*.* | *Adapted text in the Discussion, page 17:* Fourthly, there might be less to gain from ACP in some countries and contexts compared to others. For instance, some studies suggest that the US may be more prominent in its use of expensive, resource-intensive services at the end of life (41). Hence, ACP might be more effective in the US to counteract potentially futile treatment. This hypothesis warrants further study. |
|  |  |  |
| Finally, I would suggest that the conclusions do reflect this critial discussion of the study results. | We have now expanded the critical reflection of our study results. | *Discussion, page 18:* We conclude that in our large trial in European patients with advanced cancer we did not find effects of the ACTION Respecting Choices ACP intervention on quality of life, coping, patient satisfaction or shared decision-making. Potential explanation relate to characteristics of the intervention, patients’ preferences regarding ACP, the European context, and the choice of outcome measures. The increase in use of specialist palliative care and of inclusion of advance directives in hospital files is meaningful and requires further study. Our findings suggest that additional approaches to support patient-centred end-of-life care and to improve quality of life in this population are needed. |
|  |  |  |
| MINOR COMMENTS |  |  |
| Background |  |  |
| I may be wrong but I don't think I understand in how far the first 8.5 lines provide a background for this study given that ACP, as defined also by the authors in the following lines, is not an instrument to improve palliative care. | We have now shortened this introduction. | We removed the first six lines of the introduction. |
|  |  |  |
| METHODS |  |  |
| The intervention, first paragraph, line 4: What does the number in brackets (14) stand for? Reference # 14 seems not pertinent. | We thank the reviewer for noting this. Indeed, this number should be removed. | *Methods:*  we removed “(14)“ |
|  |  |  |
| RESULTS |  |  |
| Intervention delivery:  Given the low increase of written advance care plans in hospital charts, it would be interesting to add how many of the 396 patients who had ACP conversations came out of these conversations with written advance care plans. On page 8 (second paragraph) there is an information that „in the intervention group, 147 patients provided their facilitators with a copy of their completed MP forms." Since written advance care plans were one of three intervention components (#2), I would expect them to be reported here, as plain number and proportion. | We don’t know how many of the 396 patients who had ACP conversations ultimately completed advance care plans. We know the number is at least 147, which is 37% of 396 patients in the intervention arm who had at least one ACP conversation. We have now added this information to the results section. | *Results, page 11*:  In the intervention group, 147 of 396 (37%) patients who had ACP conversations provided their facilitators with a copy of their completed My Preferences form. |
|  |  |  |
| In order to further help appreciate why hospital admission was not reduced by the intervention, it would be interesting to know how many patients opted for comfort-focused care in section D of their MP form. Were the MP forms analysed by preference? | In an earlier study these forms have been analysed. We now added some of the results to the discussion section. | *Discussion, page 16:*  In a content analysis of 123 My Preferences Forms, it was found that 43.9% of patients opted for comfort-focused care only. 73% preferred home as the final place of care, 20% hospice and 10% hospital (3). |
| Primary and secondary outcome measures:  I don't think the reference to the MICE procedure needs to be repeated here. | We have removed the second reference to the MICE procedure. |  |
|  |  |  |
| **Reviewer #3:** |  |  |
| This is an interesting manuscript about a randomized control trial of advance care planning in patients with advanced cancer. The study's strengths are that it is a large study with 23 sites and is well designed with a cluster-randomized trial. However, my enthusiasm was dampened by several issues. I have the following comments and suggestions: | We thank the reviewer for these comments. |  |
|  |  |  |
| 1. In the background, the authors state that studies of ACP in patients with cancer are scarce. This is not a true statement. There are >1000 pubmed citations with "Advance Care Planning" and "Cancer". Furthermore, the authors cite several papers in their introduction. Please remove or edit this statement. | We stated that studies of ACP in patients with *advanced* cancer are scarce.  The >1000 pubmed citations rarely concern empirical studies among patients with advanced cancer. In February 2020, we identified six randomized clinical trials on effects of ACP in patients with advanced cancer.  We have now adapted our sentence. | *Abstract:*  randomized clinical trials of ACP in patients with advanced cancer are scarce. |
| 2. I am concerned about the conclusion that "Alternate approached to support patient-centered end-of-life care in the population are needed". When I think about the causal pathway, ACP is most likely to impact decisions at true end-of-life. These patients were not necessarily followed to death and there are not outcomes about the location of death or quality of decision-making at true end of life. The outcomes in this study were not impacted, so the statement should be specific to those outcomes and not all encompassing end-of-life care, which may have been measured in other ways closer to actual end of life. | Although not all patients were followed until death, 44% of patients in the intervention arm and 39% of patients in the control arm died during the study period.  Although ACP could impact decisions at the end of life, such as location of death, it could also impact decisions about for instance pursuing additional chemotherapy or not.  We have adapted the statement in the conclusion and it now refers to ‘additional approaches’. | *Discussion, page 18:* Our findings suggest that additional approaches to support patient-centred end-of-life care and to improve quality of life in this population are needed. |
| 3. I have a significant problem with the statement that "When cure is no longer possible, a focus on prolonging life is associated with worse quality of life." When treating metastatic, incurable cancer, we are most often treating with medications that are intended to BOTH prolong life and improve quality of life. While there are times when giving additional chemotherapy at end-of-life is both unlikely to prolong life or to improve quality, this statement is inaccurate the way it is written. | We agree with the reviewer and therefore have removed this statement. |  |
| 4. Please define the abbreviation ACP in the first use in the introduction. | We have now defined the abbreviation in the first use. | *Introduction, page 5:* Advance care planning (ACP) enables individuals…. |
| 5. Please clarify who was approached, who delivered the intervention. | Eligible patients were approached in participating hospitals by hospital staff.  The intervention was delivered by healthcare professionals, who were certified to deliver the ACTION Respecting Choices ACP intervention. About 50% of these healthcare professionals were involved in patient care for ACTION study participants. | *Methods, page 7:* When a care team considered patients eligible, they were asked to consider participation in ACTION. Interested patients were provided with more information about the study by the researchers.  *Methods, page 7:* Healthcare professionals, who were certified to deliver the ACTION Respecting Choices ACP intervention, used scripted conversation guides …. |
| 6. I am not clear on what the authors mean by the statement that the RC instructors followed the RC First and Advanced Steps training programme together. Please clarify. | We have now adapted this statement. | *Methods, page 6:*  We developed and evaluated the ACTION Respecting Choices (RC) ACP intervention. This was an adapted and integrated version of two of the three stages of the RC facilitated ACP conversations (First Steps and Advanced Steps). The ACTION RC ACP intervention includes three components (see S2 Text for more details). |
| 7. Please provide more detail on how many conversations were audio-recorded and were these practice conversations vs. conversations with patients with cancer. It would be important to know how the fidelity checklist was used. Did the facilitator have to get all on a certain number of times? This needs much more detail. | Conversations with ACTION participants who all had advanced cancer were audio-recorded.  We now expanded the description in the manuscript. | *Methods, page 7:* Per facilitator, fidelity checks were conducted twice; once halfway the inclusion period and once towards the end of the inclusion period. ACP conversations were audio-recorded and facilitators’ compliance with the conversation was systematically evaluated by local ACTION RC instructors, using a pilot-tested fidelity checklist, which covered the key elements of the conversation guides. |
| 8. I would like to see more information on the missingness of the data to ascertain if the multiple imputation is appropriate. Please provider a supplemental with the characteristics of those who did not complete the surveys, as this is common as people approach end of life and may impact interpretation of results. Also, please do a sensitivity analysis with a complete case. | We added in Supplementary Material a table with the distribution of lost to follow-up both at follow-up 1 and 2 by socio-demographic and clinical variables, and a comment on the multiple imputation procedure.  Given the amount of missing data and given that missings were not completely at random (Little test p < 0.0001), a complete case analysis would give largely biased results. In addition, the results of the distribution of variables by missingness suggest a missing at random assumption, which is the basis of multiple imputation. In our opinion, the complete case analysis is not appropriate with this data. We performed anyway a complete case analysis as suggested by the reviewer (Table A. at the end of this document), but we prefer not to present the results given that they are presumably highly biased. | See Supplementary material and table A. at the end of this document. |
| 9. There is a large number of people who did not consent. This should be discussed. Also, the recruitment rate for the intervention was much lower than for the control, which doesn't make sense to me in a randomized study. Please explain this finding. | In general, response rates for this kind of trials with patients with advanced cancer tend to be low.  Given the cluster-randomized nature of this study, and since hiding the nature of the intervention to participants was not ethically appropriate, patients knew beforehand whether they would receive the intervention or not. Information materials for the control group also referred in some detail to the intervention. This may have impacted the numbers of people who provided consent. |  |
| 10. It is interesting that the authors state that they did not expect survival differences and yet 44% of people in the intervention died and only 39% in the control group died. This should be evaluated further, and particular attention paid to how this would impact the survey results in terms of missing data and QoL trajectories given that patients typically have a decline in QoL before death. I am not convinced by the P values provided given the small sample size and a clinically meaningful difference. | The reviewer is right about this. This difference in mortality rates may suggest that patients in the control arm were relatively more healthy. This could be related to the differences in recruitment rates. We now refer to this difference in the discussion. | *Discussion, page. 16:* These factors may have resulted in unmeasured baseline differences between the study groups, which may also explain the fact that mortality rates were higher in the intervention than in the control arm. |
| 11. I am concerned about the population differences, in particular lung vs. colorectal cncer between groups. The higher lung cancer in the intervention could results in lower quality of life and the shorter survival in this group given that these diseases have very different prognosis. These groups should be analyzed separately given the lack of balance in the randomization. | We applied a multilevel regression model that adjusted for baseline level of the outcome measure, country and other individual characteristics, by first selecting the variables associated with emotional functioning on the basis of the Akainke’s Information Criterion: cancer stage and type showed a nonsignificant association. We added the results of this analysis in the supplementary material.  In addition, separately analyzing per cancer type does not seem appropriate, given that the power analysis was based on the overall number of participants. |  |
| 12. Please provide explanation of why 10% of the intervention did not receive the ACP. This seems high. | This was related to patients’ rapid functional decline, or to patients preferring to finalize their treatment before engaging in the ACP conversations and then becoming too ill to participate. These relatively high drop-out rates occur more often in palliative care populations (4). |  |
| 13. There should be more detail on how death was incorporated into the analysis. | We excluded deaths from the analysis because we found no significant differences in the proportion of deaths by arm, and because these proportions were small, especially at follow-up 1 (see Results, Primary and secondary outcome measures). We now highlight in the limitations that we could have performed survivor average causal effect models or partly conditional inference, and why we did not consider that approach necessary. | *Discussion page 16*:  A further limitation is that analyses were carried out on survivors only, whereas survivor average causal effect models or partly conditional inference could be a more sophisticated approach to address truncation by death (5,6). However, in our data no significant differences were found in the proportion of deaths between intervention and control arm and these proportions were small, especially at follow-up 1. |
| 14. I struggled with the outcomes selected for the study. Please provide some context as to why and how the authors anticipate that an advance directive conversation would be expected to impact quality of life and emotional functioning at 3 and 6 months if this is not associated with end of life. I recognize that the authors cannot change the data or timing now, but it would be very helpful to understand their thinking in the design and why this was designed in this way. It might even be helpful to provide a conceptual model of impact. I do understand the decision-making outcomes. | Quality of life is a multi-dimensional concept, entailing physical, emotional and social aspects.  Our primary outcome is emotional functioning, as assessed with a subscale of the EORTC-QLQ-C30 quality of life scale.  When opting for emotional functioning as the primary outcome measure of ACTION, we reasoned that an increased awareness of patients that their preferences matter, and that sharing these with relatives and healthcare professionals and knowing they would be taken into account would be very reassuring for seriously ill people.  Also, ACP conversations might shape preferences for ongoing treatment. For instance, patients might opt for more conservative treatment to avoid disabling side effects of treatment and to maximise quality of life, make the most of remaining time, rather than strive to extend it. |  |
| 15. If possible, provide an explanation of the reason why the one hospital enrolled no patients. | The one hospital experienced a series of organizational issues, which were not foreseen at the start of patient inclusion. For example, the management board changed three times during the inclusion period, which required introducing the study repeatedly. Bad experiences in another EU study ultimately made the hospital decide not to enroll patients. |  |
| 16. Please provide information available of why 13 patient did not complete any questionnaires. | Unfortunately, we have no information on why 13 patients did not complete questionnaires. |  |
| 17. Was there an element that was commonly missed in terms of the fidelity? What were the key domains evaluated? | Elements concerning fidelity that were sometimes missed related to the content of the topic guide (for example, questions about CPR not asked), the use of inappropriate communication (for example, making assumptions), or the facilitator inappropriately extending their role in the conversation to giving advice. Key domains that were evaluated related to core concepts of ACP, e.g. patient’s understanding of their own health, the completion of an advance directive, the appointment of a patient representative. |  |
| 18. The amount of imputed data should be reviewed by a statistician. | The analyses with imputed data have been conducted by an experienced statistician (GC). |  |
| 19. It is interesting that the decision-making self-efficacy, information provided by nurses/doctors, and rating of care received all declined from baseline. This could warrant comment in discussion. | Indeed, these scores all declined from baseline in both groups. We added a comment about this in the discussion section. | *Discussion, page 16:* In both intervention and control arm self-efficacy in decision-making, information as provided by doctors or nurses, and the rating of care as received declined from baseline. This unexpected finding could potentially be explained by increased awareness through study participation of the complexity of decision-making and information provision. |
| 20. I am very confused by the recruitment rate in the discussion. If the randomization is at a site level, the some received standard of care and it is unclear why they declined. Please provide some literature review of why this might have occurred and/or participation in these types of trials. | Standard of care was indeed offered in control hospitals. Participation entailed the completion of questionnaires. Given that these patients were seriously ill, it is possible that patients did not want to participate because of the burden of completing questionnaires.  We now added a reflection on this topic to our discussion. | *Discussion, page 16:*  A systematic review of recruitment issues in palliative care randomized controlled trials identified a number of barriers to recruitment that appeared to be issues in this trial. These include patients not being interested in the intervention, the burden of illness and gatekeeping by health care professionals. We also know from the general literature that health care professionals struggle to introduce ACP and discuss end of life care issues. Further research is required to explore the complexity of recruitment in palliative care trials (7). |
| 21. When the authors discuss the limited effectiveness, they should include some other studies and references to put their findings into context. The discussion should include references where they are making statements about the countries policies as well to verify legitimacy. | In our discussion of the effectiveness, we have now better contextualized our findings. | *See various parts in the discussion.* |
| 22. The discussion of Europe s. US is troubling given that these are strong statements without any literature backing them. | We agree with the reviewer that we may have been somewhat firm in our reflections on the potential lesser need for ACP in Europe. We have now adapted this section.*.* | *Adapted text in the Discussion, page 17:*  Fourthly, there might be less to gain from ACP in Europe compared to others. For instance, some studies suggest that the US may be more prominent in its use of expensive, resource-intensive services at the end of life (8). Hence, ACP might be more effective in the US to counteract potentially futile treatment. This hypothesis warrants further study. Moreover, to a certain extent, ACP may have been part of usual care in some of the countries, which may have further limited the effects of the intervention. |
| 23. I again think the conclusions of alternate approaches is not supported fully by this data, rather that the endpoints chosen are not likely at that time to be impacted by AD. | We added a reflection about our outcome measures to the Discussion section. | *Discussion, page 17:*  Secondly, the choice and timing of outcome measurements may have been suboptimal. Our primary outcome measure was quality of life, operationalized by the EORTC emotional functioning items, at 11-12 weeks of follow-up. In 2017, a large international Delphi panel agreed that quality of life is not the most appropriate outcome of ACP (6). Goal concordant care might have been a better primary outcome, but is very difficult to measure because a validated measure to assess goal-concordant care is lacking (1). Effects of ACP could also predominantly occur in the relational domain: patients and relatives may indeed have been supported to discuss and exchange views about values, goals and preferences, but this outcome was not explicitly assessed in our trial. The qualitative data as collected in the ACTION trial may shed some light on these potential effects. Although we checked medical files up until death for some patients, the timing of our outcome measurement may have been suboptimal: effects may occur at a later stage for at least some patients, at a time when their situation deteriorates and actual decisions have to be made. |

Table A. - Results from complete case analysis.

|  | **Follow-up 1 versus Baseline** | | | | **Follow-up 2 versus Baseline** | | | |
| --- | --- | --- | --- | --- | --- | --- | --- | --- |
|  | ICC | Intervention group (n=412) | Control group (n=620) | p-value | ICC | Intervention group (n=385) | Control group (n=566) | p-value |
| **Outcomes** |  |  |  |  |  |  |  |  |
| **Emotional functioning (EORTC EF-10)*** | 0.01 | -0.27 | 0.54 | 0.99 | 0.00 | 23.81 | 24.58 | 0.13 |
| **Quality of life & symptoms (EORTC QLQ-C15-PAL)** ^ | 0.04 | -2.74 | 0.25 | 0.28 | 0.03 | 6.94 | 8.82 | 0.31 |
| Overall Quality of life |  |  |  |  |  |  |  |  |
| Emotional functioning | 0.01 | 0.14 | 1.48 | 0.66 | 0.00 | -6.02 | -2.80 | 0.54 |
| Physical functioning | 0.02 | -0.66 | -1.38 | 0.78 | 0.00 | 3.57 | 2.44 | 0.13 |
| Pain | 0.01 | 1.95 | 0.64 | 0.50 | 0.01 | 55.85 | 54.71 | 0.02 |
| Dyspnoea | 0.04 | 2.32 | 1.29 | 0.43 | 0.02 | 50.10 | 51.78 | 0.81 |
| Insomnia | 0.05 | -3.86 | -1.38 | 0.16 | 0.02 | 48.33 | 46.66 | 0.86 |
| Appetite loss | 0.03 | 1.24 | 1.10 | 0.89 | 0.02 | 56.16 | 54.94 | 0.53 |
| Constipation | 0.01 | -1.72 | -0.94 | 0.64 | 0.02 | 56.15 | 57.53 | 0.51 |
| Fatigue | 0.02 | 2.35 | 0.34 | 0.64 | 0.01 | 36.51 | 35.60 | 0.21 |
| Nausea/vomiting | 0.00 | -0.36 | 0.15 | 0.85 | 0.00 | 65.56 | 64.55 | 0.33 |
| **Shared decision-making (APECC**) | 0.09 | 0.43 | 1.01 | 0.50 | 0.09 | -4.07 | 0.80 | 0.13 |
| Decision making self-efficacy scale |  |  |  |  |  |  |  |  |
| **Patient Involvement** | 0.02 | 4.25 | 0.12 | 0.13 | 0.00 | 1.63 | -2.13 | 0.04 |
| **Coping (COPE)** | 0.04 | -2.23 | -0.78 | 0.48 | 0.07 | 50.63 | 51.56 | 0.58 |
| Denial |  |  |  |  |  |  |  |  |
| Acceptance | 0.07 | -0.94 | -0.78 | 0.27 | 0.03 | -1.18 | 1.88 | 0.71 |
| Problem focused | 0.07 | -1.67 | -1.70 | 0.65 | 0.07 | 4.03 | 4.94 | 0.79 |
| **Satisfaction with care (EORTC IN-PATSAT)**^ | 0.06 | -1.51 | -1.18 | 0.57 | 0.06 | 1.72 | 0.23 | 0.34 |
| Information provision by doctors |  |  |  |  |  |  |  |  |
| Information provision by nurses | 0.04 | -0.37 | -1.05 | 0.57 | 0.04 | 1.40 | 3.35 | 0.11 |
| General rating of received care | 0.08 | -1.93 | -2.40 | 0.57 | 0.07 | -3.36 | -1.26 | 0.32 |

REFERENCES

1.	Bernacki R, Paladino J, Neville BA, et al. Effect of the serious illness care program in outpatient oncology: a cluster randomized clinical trial. *JAMA internal medicine.* 2019;179(6):751-759.

2.	Sudore RL, Fried TR. Redefining the “planning” in advance care planning: preparing for end-of-life decision making. *Annals of internal medicine.* 2010;153(4):256-261.

3.	Zwakman M, van Delden JJM, Caswell G, et al. Content analysis of Advance Directives completed by patients with advanced cancer as part of an Advance Care Planning intervention: insights gained from the ACTION trial. *Supportive Care in Cancer.* 2020;28(3):1513-1522.

4.	Preston NJ, Fayers P, Walters SJ, et al. Recommendations for managing missing data, attrition and response shift in palliative and end-of-life care research: part of the MORECare research method guidance on statistical issues. *Palliative Medicine.* 2013;27(10):899-907.

5.	Tchetgen Tchetgen EJ. Identification and estimation of survivor average causal effects. *Statistics in medicine.* 2014;33(21):3601-3628.

6.	Wen L, Terrera GM, Seaman SR. Methods for handling longitudinal outcome processes truncated by dropout and death. *Biostatistics.* 2018;19(4):407-425.

7.	Dunleavy L, Walshe C, Oriani A, Preston N. Using the ‘Social Marketing Mix Framework’to explore recruitment barriers and facilitators in palliative care randomised controlled trials? A narrative synthesis review. *Palliative medicine.* 2018;32(5):990-1009.

8.	Bekelman JE, Halpern SD, Blankart CR, et al. Comparison of site of death, health care utilization, and hospital expenditures for patients dying with cancer in 7 developed countries. *Jama.* 2016;315(3):272-283.
