# Supplementary material for: Advance care planning in patients with advanced cancer: A 6-country, cluster-randomised clinical trial
Source: PLoS Med. 2020 Nov 13;17(11):e1003422. doi: 10.1371/journal.pmed.1003422 (PMC7665676; doi:10.1371/journal.pmed.1003422)
Supplement: Attachment — Submitted filename: Response to editors & reviewers.docx [file pmed.1003422.s008.docx]

**PMEDICINE-D-20-00632R2**

| REVIEWERS” AND EDITORS” FEED BACK | AUTHORS’ RESPONSE | EDITS TO THE MANUSCRIPT |
| --- | --- | --- |
| **Requests from the editors:** |  |  |
| Please adapt the title to fit PLOS Medicine style and replace the “;” with “:” | We have adapted the title as requested. | Cover page:  Advance care planning in patients with advanced cancer: a six country, cluster-randomized clinical trial |
| Is ACTION an acronym? Please introduce on first view. The same goes for EORTC | Yes, ACTION is an acronym; Advance Care planning: an innovative palliative care intervention To Improve quality of life in ONcology patients.  Since it is quite lengthy we have now introduced it on first view in the manuscript rather than in the abstract.  We fully introduced ‘EORTC’ in the abstract. | Methods, page 5: Advance Care planning: an innovative palliative care intervention To Improve quality of life in ONcology patients (ACTION)  Abstract, page 3:  European Organization for the Research and Treatment of Cancer (EORTC) |
| Please provide participant demographics | In Table 1 we provide information on participants’ age, gender, years of education, whether they lived with a spouse or not, whether they had children, whether they considered themselves religious or not, whether they considered themselves member of a minority group, and country of residence. In addition, we indicate their diagnosis (lung or colorectal cancer) including its date and stage of cancer, and we indicate the WHO performance status. |  |
| Please temper conclusions by including “our results show” or similar | We now added ‘our results show’ to the conclusions in the abstract.  The discussion section in the paper already contained a similar statement. | Abstract, page 3: Our results show that quality of life effects were not different between patients who had ACP conversations and those who received usual care. |
| Please can you provide 95% CI along with p values as needed, when quantifying the main results | We have now provided 95% CI with p-values. | See Table 2 in the manuscript, page 11. |
| The data statement needs to be modified as authors cannot be sole contacts for data requests as per PLOS data policy. Please provide institutional or research ethics committee details for data requests. | We now provide the contact details of our department. |  |
| The author summary needs to be in bullet points | We revised the author summary, which is now in bullet points. |  |
| References must be in square brackets and bibliography in Vancouver style please | We have adapted the references as requested. |  |
| **Methods** |  |  |
| Please include your trial registry early in the methods section | We now mention the trial registry early in the methods. | Methods, page 5: The trial was registered in the International Standard RCT Number registry (ISRCTN63110516) per 10/3/2014. |
| Please can you fill the CONSORT reporting guideline and provide this as Supplementary information. Early in the methods section please state that the study has been reported according to CONSORT. Please do not use page numbers when completing the checklist. | We have filled the CONSORT reporting guideline and provided this as Supplementary information. We have stated early in the methods section that we report the study according to CONSORT and we have removed the page numbers from the checklist. | Methods, page 6:  We report the study according to CONSORT reporting guidelines, see Figure 1 Consort flow diagram. |
| Please specifically provide details of informed consent and how it was received from participants | We now provide more details of informed consent. | Methods, page 5, 6  When a care team considered patients eligible, they were asked to consider participation in ACTION. Patients who wanted to consider participation were contacted by the research team and provided with more information about the study. Patients in the intervention hospitals received information about the intervention. Those in control hospitals were informed that ACTION focused on preparing patients for decision-making about care, and that they would receive usual care. Patients were given unrestricted time to consider participation and were informed that they were free to withdraw from the study without any effect on their care. Patients who provided written informed consent were included and followed until 12 months after inclusion. |
| **Discussion** |  |  |
| Some sections require revision, in line with comments from Ref 2. There are several study design limitations that could have led to the results and therefore it would be cautious to avoid sentences such as “Fourthly, there might be relatively little to gain from ACP in Europe”. | We have now removed the sentences about the US-Europe comparison. |  |
| Please add a strengths and limitations section, to discuss the various limitations of your work that could have influenced the findings | We now more clearly indicate the strengths and limitations section in our manuscript. | Discussion, page 15. |
| The role of the funder and conflicts of interest should be reported in the article meta-data only and removed from the main manuscript document | We removed the information about the role of the funder and conflicts of interest from the main manuscript document. |  |
| Specifically, some of the comments in Reviewer 2’s notes are pertinent and require significant revision to your discussion section: “What I am saying is: From the perspective that ACP requires institutional and regional implementation, the ACTION trial’s intervention was never likely to become effective because it lacked two out of three essential components. I understand that for the authors this is a difficult position to report, and the authors do not have to share this view, but since they cooperated with Respecting Choices, and report a negative result, in my eyes it is inevitable to make this possible severe objection clear and transparent in their discussion. Limiting the objection to „countries not familiar with ACP“, in contrast, not only misses but obscures the point”. | We have revised this discussion section.  Please also see below for our responses to Reviewer 2. |  |
| “That ACP may prepare for discussions of oncologic treatment is an interesting hypothesis that has no evidence base for this special patient population, certainly not in the paper of Rebecca Sudore et al. quoted by the authors (reference #10). | We have removed the hypothesis. |  |
| In my eyes it should be discussed in the limitation section that the ACTION trial may have ended with a negative result because ACP may not be effective in changing quality of life and treatment decisions in the population of patients with advanced cancer since these rarely require treatment decisions to be based on advance care planning”. | We kindly refer to the section below (page 9), where we provide Reviewer 2 with our considerations on why ACP may be effective in patients with advanced cancer. |  |
| “In the light of what can be said about possible shortcomings of the study design (including intervention and target population), this is not a „hypothesis that warrants further study“ but a hypothesis that does not warrant to be made here” | We have removed the paragraph on the US-Europe comparison including the statement about the hypothesis warranting further study. |  |
|  |  |  |
| **Comments from the reviewers:** |  |  |
| **Reviewer #1**: Alex McConnachie, Statistical Review |  |  |
| I thank the authors for considering my original comments. In general, I am happy with their responses, and have no major concerns. | We are happy to hear this. |  |
| One comment on the sample size reduction, though. The original calculation was based on detecting a difference of half a standard deviation, or an effect size of 0.5. What is that on the scale of the EF4? Given the association between EF4 and EF10, what would be an "equivalent" difference be for the EF10? Then, given the SD of the EF10, what effect size would that equate to? | The exact size of half a standard deviation on the scale of the EF4 depends on the results in the specific patient groups as included in a study.  Please find below the explanation of the relative validity of EF10 as compared to the EF4 as given in our publication in Value in Health (Jabbarian et al, 2018):  Since the relative validity (RV) is calculated as RV= (t^2 (EF10))/(t^2 (EF4)), if RV=1.21 for the E10 vs the EF4, it means that the t-test for the EF10 was (√1.21=1.1) 1.1 times that of the EF4, or equivalent with SD(EF10)= SD(EF4)/1.1. We used this SD-ratio to calculate an estimate of the required sample for the EF10 compared to the EF4. As an example, if the EF4 with N=128 had power=80% at α=5% to detect a specific difference, then it can be calculated from the SD-ratio using standard sample size calculations that the EF10 would need N=106 to obtain the same power, or 106/128=83% of the sample for the EF4.  We used power=80%, α=5% and effect size (ES)=0.5 in the calculations. The expected savings for any combination of power and ES will be similar to those presented here, except in very extreme cases with very low power or large ES. |  |
| Also, it is notable that the ICC observed for the EF10 was smaller than was allowed for at the design stage. This is a good thing, as it should give more power, but does not seem to have been commented on. | We thank the reviewer for pointing this out and have now added a comment and a reference to this in our manuscript (discussion). | Discussion, page 15: The ICC as observed for the EF10 turned out to be smaller than was allowed for at the design stage, thus enhancing the trial power (Donner A, Klar N. Design and Analysis of Cluster Randomization Trials in Health Research. Arnold; London: 2000) |
|  |  |  |
| **Reviewer #2:** Please see below. |  |  |
|  |  |  |
| **Reviewer #3**: Thank you for addressing the comments, well done. | We are happy to hear this. |  |

**Reviewer #2:**
Thanks to the authors for considering many of my and the other reviewers‘ suggestions. In particular, I am very happy that the authors have changed „alternative“ to „additional“ in their last sentence.

***Authors’ reply****: we thank the reviewer for taking the time to thoroughly comment on our revised manuscript and rebuttal.*

In my eyes, however, some of the critical items have not been appreciated sufficiently:
**R1.1 I wrote in my review:
A. Reasons related to the intervention**
…

2.There is another important influential factor that warrants discussion. Implementing an ACP program such as Respecting Choices should not be limited to establishing a conversation process between facilitator, patient, and significant others; besides this personal level, there is also an institutional and regional level that logically must be considered if the ultimate goal of ACP that patients‘ preferences are known and honored is to be achieved.

In particular, Respecting Choices is known to have always pushed both levels, the „systems change“ level (institutional / organisational and regional development), and the personal conversation level. The ACTION trial has limited the intervention on the mere personal level. This needs to be discussed here: Why was the decision made? What were the personal, institutional and regional barriers observed in the respective countries? Are there any further indications that a „systems change“ would have been necessary to let the intervention develop a positive impact on the clinical encounters?

The authors reply,

Implementation at institutional and regional level as well as at the patient level is typical for the Respecting Choices approach. We acknowledged from the start of the ACTION project that, given the context of a clinical trial, we were not able to implement the intervention at the institutional and regional level: ACP conversations were offered only to trial participants. We could therefore not e.g. advertise in e.g. elevators or waiting rooms. This decision was thus based on methodological barriers. We indicated in the discussion that, as a result, the programme might not have been sufficiently adapted to local circumstances and needs. We now added that this was especially true for countries not familiar with ACP, such as Denmark, Slovenia and Italy, and that the programme was therefore probably not fully integrated with routine services which may have reduced its effect

and add to their discussion (p17) the sentence:

…, especially in countries not familiar with ACP, such as Denmark, Slovenia and Italy. Therefore the programme was probably not integrated with routine services which may have reduced its effect.

However, this amendment misses the decisive point. The authors concede very clearly and correctly, that „implementation at institutional and regional level … is typical for the Respecting Choices approach.“ The reason why RC has chosen to push institutional and regional implementation of ACP with the same determination as the ACP conversation itself is, however, that **ACP is not likely to be effective** without what RC calls a systems change at institutional and regional levels. Thus, it is not only „countries not familiar with ACP“ that require regional and institutional implementation of ACP in order for it to become effective; it is every group that undertakes to implement ACP at all, including RC itself in the La Crosse region. At a national ACP conference in La Crosse in autumn 2016, RC staff reported that for various reasons the La Crosse coordinator position had been vacant for more than a year („ACP coordinator being the role that ensures institutional and regional coordination). As a consequence, even a program as well-established as RC in La Crosse itself for more than 20 years immediately and notably lost effect, according to the report.

What I am saying is: From the perspective that ACP requires institutional and regional implementation, the ACTION trial’s intervention was never likely to become effective because it lacked two out of three essential components. I understand that for the authors this is a difficult position to report, and the authors do not have to share this view, but since they cooperated with Respecting Choices, and report a negative result, in my eyes it is inevitable to make this possible severe objection clear and transparent in their discussion. Limiting the objection to „countries not familiar with ACP“, in contrast, not only misses but obscures the point.

***Authors’ reply:*** *The methodological reasons as given in our first reply led us to the choice only to implement the ACP programme at the personal level. We agree with the reviewer that not implementing the programme at institutional and regional level very likely reduced its effect. We would like to emphasize that we collaborated with Respecting Choices staff in designing our approach. Had we indeed been convinced that the intervention was never likely to become effective, as the reviewer suggests, we would – of course – never have conducted the study in its current form. In addition, we would kindly like to point at a previous study that found an effect of the Respecting Choices intervention without implementing it at the regional level either (Detering et al. BMJ). For future research we would recommend to explore all options for broader involvement of the institutional level.*

***Adapted text in the manuscript, page 16.***
*As a result, the programme was not integrated with routine services, nor adapted to local circumstances and needs, which may have reduced its effect.*

*For future research we would recommend to explore all options for broader involvement of the institutional level.*

**R1.2 I wrote in my review:**

**B. Reasons related to the Study Design**

…

1.…

2.But also with regard to clinical outcomes like hospitalisation: Is ACP likely to change the course of patients with advanced, non-curable cancer? Advance Care Planning, as the authors rightly quote from the EAPC White Paper, allows individuals to reflect, to plan and to document their preferences for future critical treatment decisions that may need to be taken when the now planning individual has become incapable of decision making. Any clinical effect of ACP, then, is to be expected (only) if patients are rendered incompetent at some time in the course of their illness AND if at that time medical decisions have to be taken for which the preferences planned and documented in advance can be regarded relevant. In advanced cancer, however, it would not be unexpected that most treatment decisions are made with the competent patient, while these patients become incompetent typically (i.e., with few exceptions) in the course of their dying process when any further attempts to sustain life would be futile and therefore medically not indicated (justifiable). Therefore, it would be interesting to learn whether the authors have collected any quantitative or qualitative impressions on whether medical treatment decisions had to be taken at all with study patients incapable of decision making so that an advance care plan, if present, could have become relevant. In any case, it ought to be discussed whether ACP is likely to make an effect in this patient group –unless there is an assumption that ACP indirectly also transforms care planning, i.e. the medical decision making between oncologists and competent, actively participating patients. Such an assumption, however, should be made transparent in the discussion.

The authors reply,

Indeed, ACP is considered to enable individuals to reflect, discuss, and document preferences for future treatment and care. For a number of reasons we think this process could affect the decision-making process of patients with advanced cancer.1. ACP is conceptualised as to be especially useful for situations in which the individual has become incapable of decision making. This can also include decision-making while being in pain or in distress, as is known to be common in patients with advanced cancer. 2. The process of reflecting and discussing preferences for care can also prepare and empower patients for decisions they make themselves, while they are competent. We do not have data about whether medical treatment decisions had to be taken with participants incapable of decision-making. We now added an assumption about the potential of ACP interventions to transform care planning.

and add in their introduction:

ACP interventions have the potential to prepare patients for decision-making when they are unable to make their own decisions; moreover they have the potential to empower patients to engage in in-the-moment decision making(2).

Again, I have to say that I am not satisfied with this reply. My point was that ACP because of its very nature and purpose is not likely to be effective in advanced cancer. When patients with advanced cancer deteriorate, there is usually ample time and opportunity to discuss the remaining options for live-sustaining treatment with them – as long as there are any. I am surprised to read that instances where ACP should become effective may also be when patients with advanced cancer are „in pain or in distress“ – this statement is not based on evidence, and it is not at all plausible. When a patient with advanced cancer is in agony, then this is certainly not the time to chose between life-sustaining versus palliative treatment options. Oncologic treatment options such as operation, chemotherapy or radiation almost always presuppose consent of a competent individual, and they will always receive what is regarded necessary for an informed consent. As a consequence, any prior ACP, while it may or may not have some preparatory effect (second argument of the authors), will usually not (with rare exceptions) guide medical decision making on whether or not to use life-sustaining measures when patients with advance cancer deteriorate. It is possible, and even likely, that whether patients with advanced cancer are comfortable and enjoy best possible quality of life strongly relates to how oncological treatment options are offered to them in the sense of an optimal process of shared decision making, and also whether they are offered optimal palliative care from early on, but not so much ACP. That ACP may prepare for discussions of oncologic treatment is an interesting hypothesis that has no evidence base for this special patient population, certainly not in the paper of Rebecca Sudore et al. quoted by the authors (reference #10).

In my eyes it should be discussed in the limitation section that the ACTION trial may have ended with a negative result because ACP may not be effective in changing quality of life and treatment decisions in the population of patients with advanced cancer since these rarely require treatment decisions to be based on advance care planning.

***Authors’ reply:*** *We would like to bring forward a number of considerations:
1. Indeed, it could be that ACP is not effective in patients with advanced cancer because there is a limited need to make treatment decisions based on ACP. Limited evidence on ACP in patients with advanced cancer was one of the reasons to conduct the ACTION study.*

*2. Still, there are reasons to believe ACP could also be useful for patients with advanced cancer. The reviewer focuses on treatment decisions while ACP can also more widely consider preferences for care. There are, for instance, discrepancies in numbers of people indicating a preference to die at home versus the number of people actually dying at home (De Roo et al. Actual and preferred place of death of home-dwelling patients in four European countries: making sense of quality indicators. PLoS ONE 2014; Beccaro et al. Actual and preferred place of death of cancer patients. Results from the Italian survey of the dying of cancer (ISDOC). J Epidemiol Community Health 2006; Meeussen et al. Endof-life care and circumstances of death in patients dying as a result of cancer in Belgium and the Netherlands: a retrospective comparative study. J Clin Oncol 2011).*

*3. Studies of e.g. A.M. The and T. Hak (The A.M, Hak T., Koëter G., & van der Wal G. (2000). Collusion in doctor-patient communication about imminent death: an ethnographic study. BMJ) have shown that patients with advanced cancer are not always aware of their prognosis and of the potential harms and benefits of treatment options. Taking time to discuss what really matters to patients and an increased awareness among relatives and health care professionals of patients’ preferences may therefore affect decision-making later on in the disease trajectory. This underlines the usefulness of ACP, also in populations of patients with advanced cancer.*

*4. The reviewer questions our reference to the paper of Rebecca Sudore (Sudore RL, Fried TR. 2010). We agree and have removed these sentences from our manuscript.*

**R1.3 I wrote in my review:**

C.„The European context“

..

Given the alternative good possible explanations reflected above for the negative results found in this study, and the strong body of evidence for overdiagnosis and -treatment (and lack of patient-centred care) also in many European countries, this paper does not seem the right place to speculate whether ACP in general may not be a necessary or useful tool for Europe altogether.

The authors now write in their paper:

Fourthly, there might be relatively little to gain from ACP in Europe. Some studies suggest that the US may be more prominent in its use of expensive, resource-intensive services at the end of life, such as ICU care (48). Hence, ACP might be more effective in the US to counteract potentially futile treatment. This hypothesis warrants further study. Moreover, to a certain extent, ACP may have been part of usual care in some of the countries, which may have further limited the effects of the intervention

I remain very unhappy and somewhat upset (confer also comment # 22 of Reviewer # 3) with the now remaining assertion that „ACP (sic!) might be more effective in the US [than in Europe] to counteract potentially futile treatment“. In the light of what can be said about possible shortcomings of the study design (including intervention and target population), this is not a „hypothesis that warrants further study“ but a hypothesis that does not warrant to be made here. Again, we are talking about ACP for patients with advanced cancer, i.e. the very target population of all for whom the lowest effect of ACP is to be expected, for the reasons given above. Besides (and not mentioned in my first review), one of the strongest (and finest) pieces of evidence of ACP was a randomised controlled trial of aged patients in an Australian hospital (Detering et al, BMJ 2010) – with Australia being certainly more anglosaxon rather than US American. My suggestion, therefore, is to dismiss the US-European comparison; if the authors remain not ready to do so, than the least would be to confine their statement to the special patient population they have studied, i.e. with advanced cancer.

***Authors’ reply:*** *We thank the reviewer for these considerations and have now removed the paragraph on the US-European comparison.*
